# Use of health services by remote dwelling Aboriginal infants in tropical northern Australia: a retrospective cohort study

**DOI:** 10.1186/1471-2431-12-19

**Published:** 2012-02-28

**Authors:** Sarah J Bar-Zeev, Sue G Kruske, Lesley M Barclay, Naor H Bar-Zeev, Jonathan R Carapetis, Sue V Kildea

**Affiliations:** 1 Centre for Rural Health, Northern Rivers; School of Public Health, Sydney Medical School, University of Sydney, New South Wales 2480, Australia; 2 School of Health, Charles Darwin University, Darwin 0909, Australia; 3 Centre for Rural Health; Northern Rivers, University of Sydney, New South Wales 2480, Australia; 4 Menzies School of Health Research, Charles Darwin University, Darwin 0909, Australia; 5 Midwifery Research Unit, Australian Catholic University and the Mater Medical Research Institute, Queensland 4010, Australia

## Abstract

**Background:**

Australia is a wealthy developed country. However, there are significant disparities in health outcomes for Aboriginal infants compared with other Australian infants. Health outcomes tend to be worse for those living in remote areas. Little is known about the health service utilisation patterns of remote dwelling Aboriginal infants. This study describes health service utilisation patterns at the primary and referral level by remote dwelling Aboriginal infants from northern Australia.

**Results:**

Data on 413 infants were analysed. Following birth, one third of infants were admitted to the regional hospital neonatal nursery, primarily for preterm birth. Once home, most (98%) health service utilisation occurred at the remote primary health centre, infants presented to the centre about once a fortnight (mean 28 presentations per year, 95%CI 26.4-30.0). Half of the presentations were for new problems, most commonly for respiratory, skin and gastrointestinal symptoms. Remaining presentations were for reviews or routine health service provision. By one year of age 59% of infants were admitted to hospital at least once, the rate of hospitalisation per infant year was 1.1 (95%CI 0.9-1.2).

**Conclusions:**

The hospitalisation rate is high and admissions commence early in life, visits to the remote primary health centre are frequent. Half of all presentations are for new problems. These findings have important implications for health service planning and delivery to remote dwelling Aboriginal families.

## Background

Australian Aboriginal people have dramatically worse health outcomes than non-Aboriginal people by every measure, and this is true for children as it is for adults [1]. Although most Aboriginal people reside in cities and regional areas, approximately one quarter live in remote communities [2]. Health outcomes for Aboriginal people in remote communities tend to be worse than those in larger rural or urban centres [3].

Aboriginal newborns have higher rates of perinatal mortality, preterm birth and low birth weight than non-Aboriginal newborns [4]. Aboriginal infants also have a higher burden of illness and hospitalisation than non-Aboriginal infants [5]. Despite improvements in perinatal mortality [6] incidence rates of certain infectious diseases continue to be among the highest in the world [7]. In the Northern Territory (NT), where Aboriginal Australians comprise 30% of the population [8], respiratory and diarrhoeal diseases are the leading causes of hospitalisation for Aboriginal infants and children [9]. This burden of illness commencing in infancy foreshadows the early onset of chronic disease [10]. Aboriginal infants from remote communities in the East Arnhem region of northern Australia are frequent users of primary health services presenting on average twice per month, mostly for upper-respiratory tract and skin infections [11].

Access to appropriate, high quality health care during infancy and indeed throughout all stages of life, is considered a basic human right [12] and essential to reducing morbidity and mortality [13], but remote dwelling Aboriginal adults have less access to health services than other Australians [14]. Barriers to access include the availability of and distance from health services, transport, English proficiency [15] and insufficient attention to the cultural needs of Aboriginal people [16]. Data on health service access and utilisation by remote dwelling Aboriginal infants are limited. Planning of health services must be informed by an understanding of service utilisation patterns, particularly at the primary level. We therefore aimed to document comprehensively the health service utilisation of a cohort of Aboriginal infants born in remote NT communities.

## Methods

### Setting

Two of the study sites were the Health Centres (HCs) in two large purposively selected remote Aboriginal communities in northern Australia, located approximately 500 km from the major urban centre, Darwin. The third study site was the regional hospital in Darwin. This is the single public hospital servicing these communities and provides comprehensive tertiary, paediatric and newborn care.

Health care in remote HCs is typically provided by remote area nurses (RANS), and Aboriginal Health Workers (AHWs), with doctors consulting patients referred to them by these staff. Onsite staff are often supported by visiting paediatricians and child health nurses. Infants requiring hospitalisation are evacuated from the community to the regional hospital, approximately one-hour flight by light airplane.

### Design and data collection

We conducted a retrospective cohort study of Aboriginal infants from these communities, following them up to 12 months of age. All Aboriginal infants born 1 January 2004 to 31 December 2006 with a gestation of at least 20 weeks or birth weight of at least 400 grams and born at the regional hospital, in hostel accommodation, in transit to hospital or in the remote community, were eligible for inclusion in the study. The study cohort was constructed through manual data linkage between community birth records from the two government operated primary HCs and medical records at the regional hospital.

Data were collected using manual review of medical records at the hospital and HCs. We collected the number of episodes and reason for health service utilisation at the HC, categorising reasons for presentation according to the local guidelines for treatment of children (see Table 1) [17]. Primary and additional reasons for each presentation were recorded; multiple presentations occurring on the same day were separately enumerated. The number of hospital admissions and reason for admission were also recorded. Hospital admissions were categorised by discharge diagnoses from the discharge summary or the medical record if the summary was not available. We also recorded admissions to the regional hospital Neonatal Nursery Unit (NNU). We only included NNU admissions that lasted 4 hours or more, reasoning that some infants transited briefly through the NNU when it was uncertain if they actually required admission. Hospital outpatient visits were not included as part of this study. Many infants receive this follow up in the remote HCs by visiting specialists and this was included as part of the HC utilisation data collection.

**Table 1:** Categorisation and recorded reason for presentation at the Health Centre

Category	Documented reason for presentation
New problem	Breastfeeding problems
	Ear symptoms
	Eye symptoms
	Fever
	Gastrointestinal symptoms
	Infant supplies: formula/food/medicine
	Injury
	Non-acute newborn reasons
	No symptoms/reason for presentation recorded
	Other feeding problems
	Other reasons
	Respiratory tract symptoms
	Seizures/other neurological symptoms
	Sepsis
	Skin symptoms
	Social reasons
	Urinary tract symptoms

Routine health check	Well baby check
	Immunisation
	Growth Action and Assessment (GAA) *
	Anaemia monitoring

Review visit	Planned follow up visit specifically requested by any HC staff or visiting medical, nursing or allied health specialists (excluding paediatricians). These visits are typically used to review infants following an acute presentation or for ongoing monitoring and management of chronic problems

Paediatric Review	On-site consultation with outreach visiting paediatrician

*GAA was a NT Government program for remote dwelling children under five years at the time of the study. It was designed to improve growth and nutritional status through monitoring of growth and anaemia and appropriate interventions

Primary endpoints were the number of primary health care episodes and hospital admissions. Person-time observed commenced at birth and ceased on the day the infant turned one year old or the date the infant died.

### Ethics

Ethical approval was obtained from the Human Research Ethics Committee of the Menzies School of Health Research and the NT Department of Health and Families. The data presented here is from a baseline study nested within the National Health and Medical Research Council '1 + 1 = A Healthy Start to Life' project. This five-year project aimed to improve maternal and infant health for remote dwelling Aboriginal families in the NT.

### Statistical analysis

Data were analysed per infant and per presentation using STATA 11.1 (TM Statcorp, College Station, Texas). Continuous data are reported as means (1 standard deviation (SD), 95% Confidence Interval (CI)) or medians (Interquartile Range (IQR)) and compared using 2 tailed *t*-test assuming unequal variances if appropriate. Dichotomous data are reported as proportions and compared using χ^2^-test. Wilson confidence intervals are reported for binomial proportions.

## Results

Four hundred fifty two births were identified. Excluded were non-Aboriginal infants (n = 26) and infants born in a hospital other than the regional hospital (n = 2). Of the 424 eligible infants, 11 (2.6%) had no community or hospital record. The final cohort consisted of 413 infants, 399 of whom had both hospital and community records, 9 had a hospital record only and 5 a community record only (all born in community and never admitted to hospital). In total, 408 infant records were reviewed at the hospital and 398 at the HCs. Birth outcome data was obtained from maternal records where infant records were unavailable.

### Birth

Ninety percent (n = 371) of the 413 infants were born at the regional hospital (inborn). Ten percent were outborn; 38 of these infants were born in the remote community, and 4 were born in transit to hospital or at hostel accommodation in the regional centre. Outborn infants had significantly lower gestational age and birth weight than inborns. Mean gestation for inborns was 37.6 weeks (SD 2.6, 95% CI 37.3-37.9), for outborns 36.2 weeks (SD 3.6, 95% CI 35.0-37.2); *p *= 0.001. Mean birth weight for inborns 2998 g (SD 629, 95% CI 2933-3062), for outborns 2726 gm (SD 837,95% CI 2477-2974); *p *= 0.008. Proportion low birth weight (LBW) (< 2500 grams) was 16% among inborns and 35% among outborns, *p *= 0.002. Proportion preterm among inborn was 19% and 36% among outborns. In total, 21% of infants were born preterm (< 37 weeks gestation) and 18% were low birth weight.

### Neonatal nursery unit admissions

Overall, one third of infants were admitted to NNU for 4 hours or more. Most frequently recorded NNU discharge diagnosis are summarised in Table 2. Infants could have multiple discharge diagnoses recorded on discharge summaries.

**Table 2:** Neonatal Nursery Unit discharge diagnoses

Discharge diagnoses	Number (%)
Preterm	61 (51%)
32-36.6 weeks	42 (35%)
28-31.6 weeks	11 (9%)
< 28 weeks	8 (6%)

Low Birth weight (< 2500)	60 (50%)
2000-2499	34 (28%)
1500-1999	7 (6%)
< 1500	19 (16%)

Presumed sepsis	29 (24%)

Respiratory illness	25 (21%)
Respiratory Distress Syndrome	16 (13%)
Transient Tachypnoea of the newborn	9 (8%)

Intrauterine Growth Restriction	13 (11%)

Diabetic mother	10 (8%)

Other maternal illness	8 (6%)

Congenital anomalies	6 (5%)

Cardiac problems	6 (5%)

Mean gestation (weeks) for infants admitted and not admitted to NNU respectively was 35.5 (SD 3.8, 95% CI 34.8-36.1) and 38.3 (SD 1.7, 95% CI 38.1-38.5); *p *< 0.001. Mean birth weight for infants admitted and not admitted to NNU respectively was 2524 g (SD 828, 95% CI 2374-2673) and 3150 g (SD 468, 95% CI 3096-3204); *p *< 0.001.

### Health centre presentations

A total of 11,224 episodes of remote health service utilisation were made by the 398 infants with a community record. The median time from hospital discharge to first utilisation of the health service was 8 days (IQR 4-19) with 96% of presentations occurring at the HC and 4% at home. Two neonatal deaths occurred following hospital discharge.

First presentations were for routine health checks (80%), acute symptoms (13%) and non-acute newborn reasons (7%).

### Frequency of presentations

Infants presented to the HC between 1 and 186 times during the first year of life, median 25 (IQR 15-38), mean 28 (SD 18, 95% CI 26.4-30.0). Infants previously admitted to NNU had on average 33 presentations (95%CI 29-37), compared to 26 presentations (95%CI 24-28) for infants not previously admitted to NNU, *p *< 0.001.

### Reason for presentation

**Figure 1:**
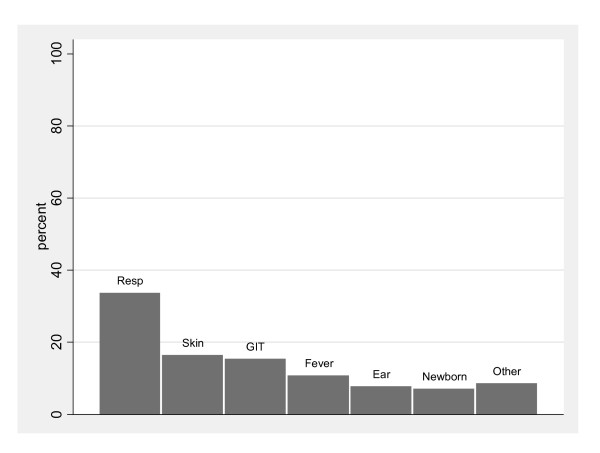
**Primary reason for new presentation to the Health Centre (excluding review and routine visits)**.

New problems were the most common reason for HC presentations (49%). These were predominantly for respiratory (resp), skin and gastrointestinal (GIT) symptoms (Figure 1). Routine health checks comprised 34% and review visits: 15% by HC staff or other visiting specialists and 2% by outreach paediatricians. Fourteen infants (3.5%) collectively had 1137 (10.1%) visits, an average of 81 visits per infant. The reasons for presentation among this group did not differ to the rest of the population.

### Age at presentation

Age at presentation was uniformly distributed throughout the first year, implying that the high rate of utilisation remained consistently high throughout the entire first year of life. In the 0-3 month age group, respiratory, skin symptoms and non-acute newborn reasons made up the bulk of reasons recorded for new problems. Presentations related to newborn reasons declined after 3 months with respiratory, skin and gastrointestinal symptoms, non-specific fever and ear symptoms dominating subsequently.

### Hospital admissions in first year

**Figure 2:**
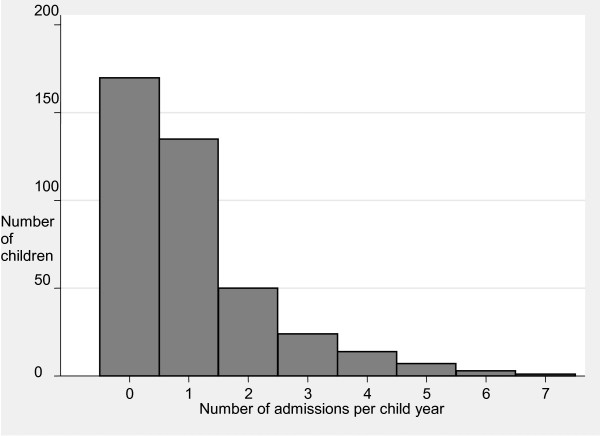
**Number of infant hospitalisations in the 1^st ^year including Neonatal Nursery Unit admissions**.

By one year of age 59% of infants were admitted to hospital at least once, the rate of hospitalisation including NNU admissions was 1.1 (95%CI 0.9-1.2) admissions per infant. The rate of admission for infants previously in NNU was more than double that among non-NNU admitted infants (*p *< 0.001). Among admitted infants, 58% had one admission, 21% two and 21% had between three and six admissions (Figure 2).

Excluding NNU admission, 47% of infants required hospital admission in the first year of life and the hospitalisation rate per infant was 0.78 (95% CI 0.70-0.88). Of the infants who were admitted to the NNU, 60% were readmitted within the first year. Overall, of the infants born preterm, 60% were readmitted compared with 44% of term babies.

**Figure 3:**
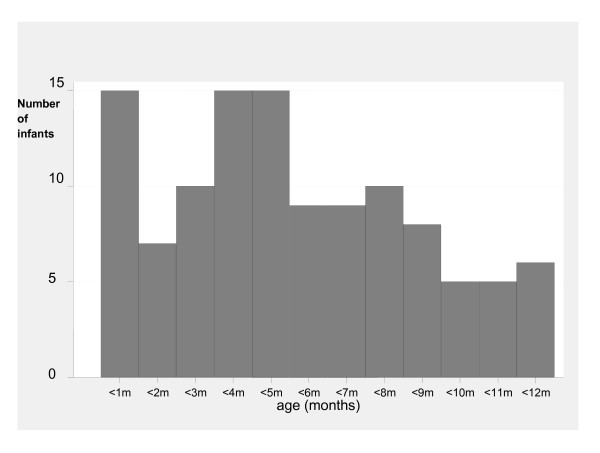
**Age at first hospitalisation in the first year of life (excluding Neonatal Nursery Unit admissions)**.

The median age at first hospitalisation excluding NNU admitted infants was 4.6 months (IQR 2.7-7.3 months) (Figure 3). Hospital admissions were predominantly for respiratory infections and gastroenteritis (Table 3).

**Table 3:** Hospital diagnosis

Diagnosis	Primary diagnosis	Secondary diagnosis
	**Total (%)**	**Total (%)**

**Respiratory infections**	145 (47%)	5 (2%)

**Gastroenteritis**	85 (27%)	2 (1%)

**Failure to thrive**	14 (5%)	6 (2%)

**Other**	12 (4%)	2 (1%)

**Skin infection**	10 (3%)	3 (1%)

**Fever without focus/Suspected sepsis**	9 (3%)	1(< 1%)

**Urinary Tract Infection**	7 (2%)	4 (1%)

**Surgery**	7 (2%)	1 (< 1%)

**Seizures**	5 (2%)	1 (< 1%)

**Anaemia**	4 (1%)	4 (1%)

**Injury**	3 (1%)	0

**None recorded**	9 (3%)	281 (91%)

**Total**	**310**	**310**

## Discussion

This study has uniquely described patterns of health service utilisation in the total infant population of two of the biggest remote communities in Australia's Northern Territory. We have documented extremely high rates of health service utilisation at the primary and referral level, commencing from birth and continuing throughout the first year. Remote-dwelling Aboriginal infants access health care frequently for both routine and acute care, despite the multiple barriers to care outlined by others [15,16].

There were a total of 11,224 presentations to the HCs for the three years of data collection. For each community, this translates to an average of 7.65 infant presentations per day (based on 249 working days in the year). The implications of this for remote workforce planning are important given that most HC presentations were for new, acute problems. The severity and complexity of many presentations in these HCs can require multiple staff to provide numerous hours of acute care to an individual infant, particularly when the infant needs emergency air evacuation to hospital. Cultural and linguistic barriers as well as staffing shortages, a lack of nurses with child health skills and qualifications and rapid turnover resulting in repeated training of new staff [18] compound this workload in remote health services. The organisation and delivery of infant health services in remote northern Australia varies across HCs. Some HCs have specific days for routine health checks by designated staff, with the acute care delivered by other staff as needed. Other communities have designated staff that delivers both routine and acute care any time that the infant presents to the HC.

Service provision is dependent upon HC funding (staffed for a 5 day week, minimal weekend service and on call service afterhours; not 24/7 service provision), availability of staff (relief not always provided for holidays or educational leave), callouts the previous night, staff skill mix and community size. Current staffing levels for infant and child health services in remote communities are not determined by their burden of disease or service usage and are insufficient to meet the needs of the young population, thus affecting the quality of care [18].

We distinguished acute care episodes from routine care at the HC. This has not been previously investigated among the remote dwelling Aboriginal infant population in the NT. We identified respiratory, skin and gastrointestinal symptoms as the leading new problems seen at the HC. Others have shown similarly high presentation rates primarily for infectious diseases in remote HCs [11]. High rates of primary health service utilisation have also been identified among suburban Victorian infants, however the bulk of the visits were unrelated to acute illness unlike our findings [19].

A third of presentations were for routine health checks and other non-acute interventions. Community based workers, Strong Women Workers and AHWs are ideally situated to provide much of this preventive care and health education in a culturally safe framework and potentially reduce the workload for clinical staff busy attending to the burden of acute illness, although this is not currently occurring in many remote settings.

Poor basic living conditions contribute to the burden of disease [20]. However, in an era when the nation is focused on closing the gap in under 5 mortality and health outcomes, providing better care for infants in their first year of life is a critical issue that must be targeted. Health services should be designed to provide high quality health care for infants as well as preventative education and effective interventions for known contributors to poor infant health outcomes such as maternal and household smoking. Ideally this should commence early in the antenatal period.

Several approaches to improving health service effectiveness are being introduced across remote communities including the Healthy Under Five Kids program, designated child and family health nurse positions, and the expansion of family support workers. These programs are in their implementation phase and have not been funded to be rigorously evaluated.

We identified a high rate of hospitalisation. One third of infants were admitted to the NNU following birth. This is double the admission rate for non-Aboriginal infants in the rest of Australia [21]. More than half of the infants admitted were born preterm. The total preterm birth rate was 6% higher in these communities compared with the preterm birth rate among other Aboriginal babies in the NT [21]. Problems with the accurate estimation of gestational age due to poor maternal recall of menstrual period dates and uptake of early ultrasound, are well described in the Australian Aboriginal population [22-24]. We identified 8/42 premature LBW whose true gestation we could calculate based on 1^st ^trimester ultrasound. Only one case of misclassification as premature occurred.

Excluding NNU admissions, 47% of infants had at least one hospital admission before they turn one. The high rates of admissions for respiratory infection identified in our study concur with other NT studies [25,26].

Despite the large number of visits audited, the retrospective nature of this study limits causal inference and a number of infant records were unavailable for review. It seems likely that these few records were missing completely at random so the impact on inference is likely to be minimal. Data linkage between primary HC and hospital records was complicated by infants with multiple first and surnames and addresses; some misidentification of infants may have occurred. Finally, given the mobility of Aboriginal populations in the NT [27], infants may have presented for care at other health services or have been admitted to a hospital other than the regional hospital reviewed in this study, in which case our results would only underestimate service utilisation.

## Conclusions

Remote dwelling Aboriginal families seek health care for their infants frequently. There have been few studies that can provide comparative data with these results. These infants have extremely high rates of health service utilisation and hospitalisation representing an appalling disease burden among this population. HCs are not staffed to provide this level of care for the under one-year population. Optimising the delivery of preventive and curative health services through targeted workforce planning and evidence based approaches, which engage families and the broader community, should be implemented and evaluated.

## Competing interests

The authors declare that they have no competing interests.

## Authors' contributions

SBZ was responsible for the study design, obtaining ethical approval, data collection, data analysis and drafting the manuscript. NBZ assisted with data cleaning and analysis. SGK, LMB and SVK participated in designing the study and provided comments on the analysis and manuscript together with JC. All authors read and approved the final manuscript.

## Pre-publication history

The pre-publication history for this paper can be accessed here:

http://www.biomedcentral.com/1471-2431/12/19/prepub
